# Supplementary material for: A Study of Greek Graviera Cheese by NMR-Based Metabolomics
Source: Molecules. 2023 Jul 18;28(14):5488. doi: 10.3390/molecules28145488 (PMC10385548; doi:10.3390/molecules28145488)

A study of Greek graviera cheese by NMR-based metabolomics

Evangelia Ralli, Apostolos Spyros*

NMR Laboratory. Department of Chemistry. University of Crete. Voutes campus. 710 03 Heraklion. Crete. Greece

* Correspondence: email: aspyros@uoc.gr; Tel:+30 2810545085

SUPPLEMENTARY MATERIAL

Table S1: Sample information including origin, year of production and maturation time.

Table S2: Mean, minimum, maximum, and standard deviation values expressed as mg/kg of cheese or % content (lipids) for all metabolites of graviera samples according to geographical origin.

Table S3: Analysis of Variance (ANOVA) of Cretan cheese samples. Grouping variable: Area of production (Chania, Rethymnon, Heraklion, Lasithi). Variables with $p<0.05$ are marked in red.

Table S4: Analysis of Variance (ANOVA) of cheese samples. Grouping variable: Area of production (Crete, Cyclades, Mainland)

Table S5: Cross validation ANOVA of the untargeted OPLS-DA model regarding cretan/non cretan origin depicted in Figure 6, right.

Figure S1: Permutation diagram of the untargeted PLS-DA model regarding cretan/non cretan origin depicted in Fig. 6, right.

Figure S2: Aliphatic region of the ^{1}H-^{13}C multiplicity-edited gHSQC 2D NMR spectrum of the polar extracts of graviera cheese at a ^{1}H frequency of 500 MHz, blue contours represent CH/CH_3 protons, green contours represent CH_2 protons.

Figure S3: Aromatic region of the ^{1}H-^{13}C multiplicity-edited gHSQC 2D NMR spectrum of the polar extracts of graviera cheese at a ^{1}H frequency of 500 MHz, blue contours represent CH/CH_3 protons, green contours represent CH_2 protons.

Figure S4: Aliphatic region of the long range ^{1}H-^{13}C gHMBC 2D NMR spectrum of the polar extracts of graviera cheese at a ^{1}H frequency of 500 MHz.

Figure S5: Aliphatic region of the ^{1}H-^{1}H gTOCSY 2D NMR spectrum of the polar extracts of graviera cheese at a ^{1}H frequency of 500 MHz.

Table S1. Sample information including origin, year of production and maturation time.

Sample	Origin	Area	Region	Year	Maturation
1	Cretan	Heraklion	Crete	2012	8
2	Cretan	Heraklion	Crete	2012	8
3	Cretan	Chania	Crete	2012	4
4	Cretan	Heraklion	Crete	2012	9
5	Cretan	Heraklion	Crete	2012	8
6	Cretan	Heraklion	Crete	2012	12
7	Cretan	Heraklion	Crete	2012	22
8	Cretan	Rethimno	Crete	2012	7
9	Cretan	Rethimno	Crete	2012	5
10	Cretan	Heraklion	Crete	2012	8
11	Cretan	Heraklion	Crete	2013	8
12	Cretan	Heraklion	Crete	2013	10
13	Cretan	Heraklion	Crete	2013	9
14	Cretan	Heraklion	Crete	2013	11
15	Cretan	Heraklion	Crete	2013	12
16	Cretan	Rethimno	Crete	2013	12
17	Cretan	Rethimno	Crete	2013	3
18	Cretan	Rethimno	Crete	2013	13
19	Cretan	Rethimno	Crete	2013	12
20	Cretan	Heraklion	Crete	2013	3
21	Cretan	Heraklion	Crete	2013	3
22	Cretan	Rethimno	Crete	2013	10
23	Cretan	Rethimno	Crete	2013	5
24	Cretan	Heraklion	Crete	2014	8
25	Cretan	Heraklion	Crete	2013	4
26	Cretan	Heraklion	Crete	2014	10
27	Cretan	Rethimno	Crete	2014	5
28	Cretan	Rethimno	Crete	2014	4
29	Cretan	Rethimno	Crete	2014	5
30	Cretan	Heraklion	Crete	2014	3
31	Non cretan	Mytilini	-	2014	6
32	Non cretan	Athens	Mainland	2013	13
33	Non cretan	Thrace	Mainland	2014	5
34	Non cretan	Naxos	Cyclades	2014	6
35	Non cretan	Tinos	Cyclades	2013	10
36	Non cretan	Amphilohia	Mainland	2013	8
37	Non cretan	Mainalo	Mainland	2013	9
38	Cretan	Chania	Crete	2014	5
39	Non cretan	Athens	Mainland	2014	7
40	Cretan	Rethimno	Crete	2014	4
41	Cretan	Rethimno	Crete	2013	15
42	Cretan	Heraklion	Crete	2014	9
43	Cretan	Heraklion	Crete	2014	10
44	Cretan	Heraklion	Crete	2014	4
45	Cretan	Rethimno	Crete	2014	11
46	Cretan	-	Crete	2014	7
47	Cretan	Rethimno	Crete	2014	6
48	Cretan	Heraklion	Crete	2014	10
49	Non cretan	Naxos	Cyclades	2014	6

50	Non cretan	Trikala	Mainland	2014	6
51	Non cretan	Arta	Mainland	2014	13
52	Non cretan	Athens	Mainland	2014	10
53	Non cretan	Tinos	Cyclades	2014	6
54	Non cretan	Ios	Cyclades	2014	13
55	Non cretan	Mytilini	-	2014	10
56	Non cretan	Amphilohia	Mainland	2014	11
57	Cretan	Rethimno	Crete	2014	5
58	Cretan	Heraklion	Crete	2017	9
59	Cretan	Heraklion	Crete	2017	8
60	Cretan	Chania	Crete	2018	9
61	Cretan	Chania	Crete	2018	10
62	Cretan	Chania	Crete	2018	10
63	Cretan	-	Crete	2018	5
64	Cretan	Rethimno	Crete	2018	8
65	Cretan	Rethimno	Crete	2018	4
66	Non cretan	Korinthos	Mainland	2018	7
67	Cretan	Lasithi	Crete	2018	10
68	Cretan	Rethimno	Crete	2018	10
69	Cretan	Heraklion	Crete	2018	8
70	Cretan	Heraklion	Crete	2019	5
71	Cretan	Heraklion	Crete	2018	8
72	Cretan	Heraklion	Crete	2018	11
73	Cretan	Lasithi	Crete	2019	6
74	Cretan	Chania	Crete	2019	6

Table S2. Mean, minimum, maximum, standard deviation values expressed as mg/kg of cheese or % content (lipids) for all metabolites of graviera samples according to geographical origin.

	Heraklion				Rethymnon			
	n=28				n=19			
	**AVERAGE**	**Min**	**Max**	**STDEV**	**AVERAGE**	**Min**	**Max**	**STDEV**
**Lactate**	15153.90	6230.08	26997.84	4605.71	13029.05	6338.41	17507.53	2983.44
**Citrate**	680.01	0.00	1572.75	493.44	313.62	0.00	1192.97	398.33
**Succinate**	375.85	0.00	1135.88	293.54	403.13	23.39	1339.31	405.60
**Acetate**	222.65	0.00	741.93	177.07	343.52	29.85	1238.04	340.20
**Valine**	1635.43	229.55	5206.79	1262.46	886.84	88.62	2883.63	685.34
**Isoleucine**	1088.42	107.29	3794.30	1006.36	465.30	60.62	2317.80	524.35
**Alanine**	910.09	61.36	2640.15	630.99	463.18	65.72	2208.88	496.98
**Threonine**	383.91	31.94	1431.51	358.77	137.90	0.00	632.46	158.28
**Phenylalanine**	1343.88	79.66	3866.02	949.63	613.19	28.74	2106.17	499.57
**Tyrosine**	340.07	21.65	1093.76	320.08	139.77	18.14	704.89	187.41
**Tyramine**	106.43	0.00	561.45	117.94	87.57	0.00	206.59	47.68
**Aspartate**	774.75	151.97	2588.00	740.31	353.64	0.00	1986.88	446.74
**Asparagine**	1140.43	0.00	3389.77	904.70	498.99	0.00	1959.84	480.02
**Glycine**	247.79	21.14	971.52	257.11	110.23	6.84	706.88	158.87
**Methionine**	761.10	100.49	2266.39	557.76	412.74	62.67	1078.70	273.01
**Proline**	2316.13	162.28	15408.10	3048.75	711.68	0.00	3620.73	870.85
**Glutamate**	3724.94	0.00	12049.15	3164.99	1487.12	0.00	5094.84	1300.15
**Lysine**	1964.04	0.00	7082.58	1640.93	1110.32	0.00	6002.18	1339.21
**Glutamine**	733.52	0.00	3704.04	876.97	194.49	0.00	752.56	213.52
**Tryptophane**	87.19	16.39	354.03	83.19	39.71	0.00	140.17	31.98
**Uracil**	29.84	6.32	65.46	13.39	20.11	1.41	49.45	11.93
**Glycerol**	247.97	0.00	913.89	232.28	130.89	0.00	312.59	92.91
**Benzoate**	16.36	5.70	34.15	6.23	12.34	0.00	25.68	5.49
**Ornithine**	574.00	0.00	2191.32	553.93	277.19	0.00	787.69	226.12
**Choline**	25.37	0.00	106.87	21.54	29.73	10.49	80.24	15.74
**GABA**	161.28	0.00	1754.25	406.93	477.93	0.00	1220.86	495.05
**Cytosine**	4.96	0.00	11.76	4.04	0.35	0.00	3.88	1.07
**Propionate**	0.03	0.00	0.78	0.15	0.28	0.00	5.34	1.22
**Butyrate**	0.78	0.00	21.16	4.00	0.12	0.00	2.37	0.54
**Galactose**	1.41	0.00	27.62	5.60	0.00	0.00	0.00	0.00
**CLA**	0.98	0.54	1.41	0.21	0.82	0.57	1.03	0.13
**caproleic**	0.39	0.20	0.57	0.08	0.50	0.38	0.59	0.06
**butyric**	8.38	6.05	9.97	0.95	7.74	5.49	9.60	1.27
**LN**	0.92	0.49	1.39	0.24	0.74	0.15	1.08	0.24
**LO**	2.33	1.74	3.31	0.39	2.62	1.61	3.77	0.54
**OL**	23.85	20.15	33.99	2.82	20.40	12.05	24.49	2.57
**SAT**	63.55	54.33	68.95	2.94	67.70	63.92	75.38	2.79
**Sterols**	0.15	0.13	0.17	0.01	0.16	0.11	0.21	0.02
**1,2-DG**	1.56	0.68	2.95	0.54	1.50	0.94	3.69	0.66
**TFAA**	17451.68	2231.17	54088.44	13785.94	7625.11	881.14	30406.89	6724.49

	Chania				Lasithi			
	n=6				n=2			
	**AVERAGE**	**Min**	**Max**	**STDEV**	**AVERAGE**	**Min**	**Max**	**STDEV**
**Lactate**	10175.76	6173.15	13022.67	2696.21	15971.97	15056.31	16887.63	1294.94
**Citrate**	65.77	0.00	309.46	124.15	934.27	878.94	989.59	78.24
**Succinate**	367.53	89.55	752.62	293.69	208.32	188.09	228.55	28.60
**Acetate**	197.64	95.23	303.12	85.67	57.61	0.00	115.23	81.48
**Valine**	865.30	537.10	1585.49	392.53	1737.03	1514.19	1959.87	315.14
**Isoleucine**	523.09	232.24	1251.04	378.93	1460.95	1218.65	1703.26	342.67
**Alanine**	578.70	230.84	983.69	255.92	1253.44	1153.68	1353.19	141.08
**Threonine**	193.93	33.97	370.39	128.52	433.99	325.47	542.51	153.47
**Phenylalanine**	625.15	289.12	1239.74	342.90	1405.11	1359.05	1451.17	65.14
**Tyrosine**	140.66	0.00	595.29	230.18	390.13	174.45	605.80	305.01
**Tyramine**	59.89	24.87	125.83	37.58	20.97	0.00	41.95	29.66
**Aspartate**	339.67	204.24	613.13	150.85	826.38	731.67	921.09	133.94
**Asparagine**	552.97	188.67	1432.26	460.41	1623.52	1418.18	1828.87	290.40
**Glycine**	128.27	57.37	262.20	73.18	339.83	316.34	363.33	33.23
**Methionine**	416.40	237.71	614.66	154.38	860.54	681.70	1039.38	252.91
**Proline**	901.79	463.64	2260.89	684.94	2302.69	2008.96	2596.43	415.41
**Glutamate**	2081.42	1208.23	3799.17	943.39	4020.59	3418.77	4622.40	851.09
**Lysine**	1210.01	501.98	2326.42	624.66	3150.61	2658.97	3642.26	695.29
**Glutamine**	242.32	0.00	717.78	266.17	1349.77	1065.04	1634.49	402.66
**Tryptophane**	34.34	20.08	59.43	14.91	68.18	44.43	91.92	33.58
**Uracil**	21.03	12.74	35.45	8.32	40.95	35.97	45.94	7.05
**Glycerol**	165.58	43.45	249.70	69.98	161.83	105.15	218.50	80.15
**Benzoate**	10.23	5.16	19.53	5.79	16.21	11.14	21.28	7.17
**Ornithine**	359.94	71.18	848.79	277.00	0.00	0.00	0.00	0.00
**Choline**	19.55	10.55	26.06	5.34	21.71	10.55	32.87	15.78
**GABA**	74.69	0.00	448.15	182.96	0.00	0.00	0.00	0.00
**Cytosine**	2.03	0.00	7.69	2.98	10.03	6.75	13.31	4.64
**Propionate**	0.00	0.00	0.00	0.00	0.00	0.00	0.00	0.00
**Butyrate**	0.00	0.00	0.00	0.00	0.00	0.00	0.00	0.00
**Galactose**	0.00	0.00	0.00	0.00	0.00	0.00	0.00	0.00
**CLA**	0.87	0.73	1.05	0.11	1.05	0.73	1.37	0.45
**caproleic**	0.56	0.42	0.66	0.08	0.50	0.45	0.54	0.06
**butyric**	8.52	6.49	9.49	1.07	9.56	8.73	10.40	1.18
**LN**	0.83	0.53	0.92	0.15	1.08	0.71	1.45	0.52
**LO**	2.50	2.22	3.14	0.34	2.29	2.07	2.50	0.31
**OL**	20.57	19.56	22.85	1.21	20.83	20.27	21.39	0.79
**SAT**	66.71	65.78	67.63	0.66	65.19	64.44	65.94	1.06
**Sterols**	0.16	0.14	0.17	0.02	0.16	0.16	0.17	0.01
**1,2-DG**	1.22	0.65	1.66	0.37	0.88	0.84	0.91	0.05
**TFAA**	8834.01	5040.18	18088.78	4879.64	21222.76	18089.55	24355.97	4431.03

	Mainland				Lesvos			
	n=10				n=2			
	**AVERAGE**	**Min**	**Max**	**STDEV**	**AVERAGE**	**Min**	**Max**	**STDEV**
**Lactate**	16851.25	11310.36	37828.97	7637.28	13643.80	12379.94	14907.66	1787.37
**Citrate**	496.03	0.00	1229.99	539.73	169.00	0.00	337.99	239.00
**Succinate**	429.51	43.77	768.18	264.37	238.25	51.49	425.02	264.13
**Acetate**	312.05	52.13	879.84	243.87	106.02	59.79	152.26	65.38
**Valine**	1433.06	354.63	2846.75	705.23	485.62	307.87	663.37	251.38
**Isoleucine**	754.96	191.51	2264.06	617.03	254.06	182.18	325.95	101.66
**Alanine**	786.75	277.27	1689.97	434.31	233.81	149.12	318.49	119.77
**Threonine**	277.77	116.37	792.05	202.52	68.79	49.36	88.22	27.48
**Phenylalanine**	1456.59	307.37	2600.44	744.68	414.10	114.38	713.82	423.87
**Tyrosine**	330.19	44.67	749.97	256.18	35.05	28.67	41.43	9.02
**Tyramine**	124.11	0.00	237.53	94.03	94.85	52.14	137.55	60.39
**Aspartate**	465.45	0.00	1476.24	474.78	154.70	104.44	204.96	71.08
**Asparagine**	961.48	362.20	2158.03	590.28	322.86	221.14	424.58	143.86
**Glycine**	776.21	39.75	5940.14	1823.76	44.80	30.36	59.24	20.42
**Methionine**	652.97	173.94	1406.38	363.84	244.50	174.78	314.21	98.60
**Proline**	1556.84	681.17	3335.64	827.63	423.32	197.70	648.94	319.08
**Glutamate**	2568.84	1173.79	5363.35	1534.57	1016.97	435.20	1598.75	822.75
**Lysine**	2033.81	944.24	3912.32	990.42	443.42	0.00	886.85	627.10
**Glutamine**	653.18	32.03	1933.03	518.70	0.00	0.00	0.00	0.00
**Tryptophane**	95.74	30.27	151.07	40.98	32.09	25.52	38.65	9.28
**Uracil**	26.94	12.33	58.55	14.05	16.79	11.97	21.61	6.82
**Glycerol**	263.85	74.27	774.91	230.36	124.20	120.93	127.46	4.62
**Benzoate**	15.81	3.62	24.90	5.91	11.99	10.14	13.85	2.63
**Ornithine**	465.75	0.00	1163.60	338.81	241.04	106.08	376.01	190.87
**Choline**	34.55	11.25	89.36	23.98	30.05	16.09	44.01	19.74
**GABA**	1348.01	0.00	4873.25	1641.61	0.00	0.00	0.00	0.00
**Cytosine**	5.40	0.00	10.56	3.34	1.84	0.00	3.67	2.60
**Propionate**	0.00	0.00	0.00	0.00	0.00	0.00	0.00	0.00
**Butyrate**	0.54	0.00	5.38	1.70	0.00	0.00	0.00	0.00
**Galactose**	0.00	0.00	0.00	0.00	0.00	0.00	0.00	0.00
**CLA**	0.73	0.36	1.48	0.33	0.69	0.68	0.70	0.02
**caproleic**	0.54	0.47	0.59	0.04	0.35	0.33	0.37	0.02
**butyric**	7.79	6.56	9.58	0.82	8.29	7.94	8.64	0.49
**LN**	0.85	0.51	1.36	0.26	0.56	0.22	0.90	0.48
**LO**	2.26	1.94	2.54	0.16	3.43	2.81	4.05	0.88
**OL**	21.28	19.09	23.41	1.50	20.93	20.60	21.27	0.47
**SAT**	67.08	64.35	69.77	1.89	66.09	65.68	66.51	0.59
**Sterols**	0.17	0.14	0.19	0.01	0.14	0.13	0.15	0.01
**1,2-DG**	1.94	0.84	3.58	0.83	1.13	1.08	1.18	0.07
**TFAA**	14803.85	5291.00	35548.55	8734.53	4174.10	2033.85	6314.36	3026.78

	Naxos				Tinos			
	n=2				n=2			
	**AVERAGE**	**Min**	**Max**	**STDEV**	**AVERAGE**	**Min**	**Max**	**STDEV**
**Lactate**	14248.58	13921.93	14575.22	461.94	13871.27	13026.78	14715.76	1194.29
**Citrate**	0.00	0.00	0.00	0.00	59.62	0.00	119.23	84.31
**Succinate**	503.04	390.47	615.61	159.20	167.77	164.77	170.77	4.25
**Acetate**	236.06	175.85	296.27	85.15	239.35	109.08	369.63	184.24
**Valine**	1251.80	1173.78	1329.82	110.34	1693.51	1646.89	1740.14	65.94
**Isoleucine**	815.42	693.11	937.73	172.97	711.32	527.49	895.15	259.97
**Alanine**	648.22	640.28	656.16	11.23	951.19	867.68	1034.70	118.10
**Threonine**	344.15	306.22	382.08	53.64	499.29	445.83	552.74	75.60
**Phenylalanine**	1260.21	1211.27	1309.15	69.21	2035.78	2004.72	2066.83	43.92
**Tyrosine**	462.92	333.25	592.59	183.38	863.61	848.06	879.17	22.00
**Tyramine**	40.93	0.00	81.87	57.89	194.43	0.00	388.86	274.97
**Aspartate**	457.84	397.29	518.39	85.63	605.85	540.61	671.08	92.26
**Asparagine**	945.84	912.27	979.41	47.48	1341.64	1295.88	1387.40	64.72
**Glycine**	339.74	312.67	366.82	38.30	465.11	439.30	490.91	36.50
**Methionine**	413.66	413.46	413.86	0.28	814.36	777.31	851.41	52.40
**Proline**	1977.51	1852.13	2102.89	177.31	2568.78	2525.20	2612.36	61.64
**Glutamate**	1292.09	244.30	2339.89	1481.81	4890.77	4504.19	5277.34	546.70
**Lysine**	2727.70	2307.64	3147.76	594.05	2956.64	2734.83	3178.44	313.68
**Glutamine**	827.60	660.99	994.21	235.62	996.80	934.17	1059.42	88.57
**Tryptophane**	32.55	0.00	65.09	46.03	168.69	165.31	172.07	4.78
**Uracil**	8.87	8.10	9.63	1.08	8.85	8.21	9.50	0.91
**Glycerol**	276.74	114.82	438.65	228.98	164.73	87.91	241.56	108.65
**Benzoate**	6.69	6.11	7.28	0.82	5.09	4.96	5.22	0.19
**Ornithine**	795.74	768.63	822.85	38.34	730.77	373.44	1088.10	505.34
**Choline**	21.22	12.13	30.31	12.85	45.21	43.68	46.73	2.16
**GABA**	1226.49	702.48	1750.49	741.06	0.00	0.00	0.00	0.00
**Cytosine**	4.96	3.71	6.21	1.76	1.62	0.00	3.25	2.30
**Propionate**	0.00	0.00	0.00	0.00	0.00	0.00	0.00	0.00
**Butyrate**	0.00	0.00	0.00	0.00	0.00	0.00	0.00	0.00
**Galactose**	0.00	0.00	0.00	0.00	0.00	0.00	0.00	0.00
**CLA**	0.51	0.45	0.58	0.10	0.54	0.48	0.61	0.09
**caproleic**	0.47	0.46	0.48	0.02	0.59	0.52	0.66	0.10
**butyric**	8.48	8.34	8.62	0.20	6.75	6.59	6.90	0.22
**LN**	0.42	0.41	0.43	0.02	0.38	0.33	0.43	0.07
**LO**	2.57	2.17	2.97	0.56	2.88	2.81	2.95	0.10
**OL**	23.63	23.39	23.88	0.35	24.33	23.43	25.23	1.27
**SAT**	64.39	63.94	64.84	0.63	65.12	64.44	65.81	0.97
**Sterols**	0.18	0.16	0.19	0.02	0.19	0.18	0.19	0.01
**1,2-DG**	2.76	1.98	3.54	1.10	2.62	2.50	2.74	0.16
**TFAA**	13797.26	13637.93	13956.60	225.33	21563.32	21527.67	21598.98	50.42

Table S3. Analysis of Variance (ANOVA) of Cretan cheese samples. Grouping variable: Area of production (Chania, Rethymnon, Heraklion, Lasithi). Variables with $p < 0.05$ are marked in red.

Variables	SS Effect	df Effect	MS Effect	SS Error	df Error	MS Error	F	p
Lactate	1.502395E+08	3	50079830	7.709802E+08	51	15117259	3.31276	0.027153
Citrate	3.074792E+06	3	1024931	9.513367E+06	51	186537	5.49453	0.002387
Succinate	7.035978E+04	3	23453	5.719689E+06	51	112151	0.20912	0.889624
Acetate	2.758332E+05	3	91944	2.973142E+06	51	58297	1.57717	0.206269
Valine	7.908772E+06	3	2636257	5.235684E+07	51	1026605	2.56794	0.064526
Isoleucine	5.757008E+06	3	1919003	3.312859E+07	51	649580	2.95422	0.041118
Alanine	2.945860E+06	3	981953	1.554328E+07	51	304770	3.22195	0.030152
Threonine	7.778985E+05	3	259300	4.032469E+06	51	79068	3.27945	0.028216
Phenylalanine	7.312406E+06	3	2437469	2.943282E+07	51	577114	4.22355	0.009637
Tyrosine	5.689648E+05	3	189655	3.756407E+06	51	73655	2.57491	0.064002
Tyramine	2.247477E+04	3	7492	4.244452E+05	51	8322	0.90017	0.447600
Aspartate	2.502645E+06	3	834215	1.852176E+07	51	363172	2.29703	0.088650
Asparagine	6.401290E+06	3	2133763	2.739087E+07	51	537076	3.97293	0.012780
Glycine	2.821408E+05	3	94047	2.267081E+06	51	44453	2.11567	0.109697
Methionine	1.728437E+06	3	576146	9.924321E+06	51	194595	2.96075	0.040807
Proline	3.326230E+07	3	11087435	2.671308E+08	51	5237858	2.11679	0.109553
Glutamate	6.276669E+07	3	20922231	3.060655E+08	51	6001284	3.48629	0.022243
Lysine	1.393843E+07	3	4646144	1.074189E+08	51	2106253	2.20588	0.098666
Glutamine	5.128467E+06	3	1709489	2.210219E+07	51	433376	3.94458	0.013196
Tryptophane	3.157540E+04	3	10525	2.075196E+05	51	4069	2.58666	0.063128
Uracil	1.667727E+03	3	556	7.801071E+03	51	153	3.63429	0.018777
Glycerol	1.640348E+05	3	54678	1.643072E+06	51	32217	1.69718	0.179272
Benzoate	2.981405E+02	3	99	1.808830E+03	51	35	2.80203	0.049087
Ornithine	1.423123E+06	3	474374	9.588714E+06	51	188014	2.52308	0.068006
Choline	5.607726E+02	3	187	1.737696E+04	51	341	0.54861	0.651360
GABA	1.515283E+06	3	505094	9.049649E+06	51	177444	2.84650	0.046608
Cytosine	3.423929E+02	3	114	5.283319E+02	51	10	11.01709	0.000011
CLA	3.289704E-01	3	0	1.817113E+00	51	0	3.07768	0.035629
caproleic	2.247457E-01	3	0	2.995449E-01	51	0	12.75493	0.000002
butyric	9.265674E+00	3	3	6.015411E+01	51	1	2.61855	0.060816
LN	4.804903E-01	3	0	2.905450E+00	51	0	2.81138	0.048555
LO	9.768583E-01	3	0	9.879828E+00	51	0	1.68086	0.182730
OL	1.574917E+02	3	52	3.419198E+02	51	7	7.83037	0.000215
SAT	2.067946E+02	3	69	3.765459E+02	51	7	9.33620	0.000051
Sterols	5.041632E-04	3	0	1.254374E-02	51	0	0.68327	0.566374
1,2-DG	1.325171E+00	3	0	1.622639E+01	51	0	1.38835	0.256924
TFAA	1.388877E+09	3	462959015	6.070246E+09	51	119024430	3.88961	0.014043
Propionate	8.407624E-01	3	0	2.755356E+01	51	1	0.51873	0.671283
Butyric	6.696764E+00	3	2	4.364988E+02	51	9	0.26081	0.853280
Galactose	2.720950E+01	3	9	8.461952E+02	51	17	0.54664	0.652663

Table S4. Analysis of Variance (ANOVA) of cheese samples. Grouping variable: Area of production (Crete, Cyclades, Mainland)

Variables	SS Effect	df Effect	MS Effect	SS Error	df Error	MS Error	F	p
Lactate	72882721	2	36441361	1.470928E+09	68	21631294	1.68466	0.193157
Citrate	319026	2	159513	1.627717E+07	68	239370	0.66639	0.516882
Succinate	29809	2	14904	6.496702E+06	68	95540	0.15600	0.855862
Acetate	69420	2	34710	3.938707E+06	68	57922	0.59925	0.552098
Valine	220701	2	110350	6.337427E+07	68	931975	0.11840	0.888519
Isoleucine	614802	2	307401	4.025057E+07	68	591920	0.51933	0.597260
Alanine	186972	2	93486	1.965176E+07	68	288997	0.32349	0.724730
Threonine	165044	2	82522	4.936834E+06	68	72600	1.13666	0.326909
Phenylalanine	2288873	2	1144437	4.181691E+07	68	614955	1.86101	0.163349
Tyrosine	846808	2	423404	5.007872E+06	68	73645	5.74924	0.004933
Tyramine	10770	2	5385	6.457556E+05	68	9496	0.56706	0.569852
Aspartate	805898	2	402949	2.220548E+07	68	326551	1.23395	0.297575
Asparagine	733190	2	366595	3.619627E+07	68	532298	0.68870	0.505696
Glycine	179805	2	89902	2.907046E+06	68	42751	2.10295	0.129967
Methionine	47850	2	23925	1.256059E+07	68	184715	0.12952	0.878730
Proline	3636816	2	1818408	3.053996E+08	68	4491171	0.40489	0.668651
Glutamate	6579619	2	3289810	4.006192E+08	68	5891459	0.55840	0.574723
Lysine	7210436	2	3605218	1.322196E+08	68	1944406	1.85415	0.164416
Glutamine	806828	2	403414	3.006697E+07	68	442161	0.91237	0.406431
Tryptophane	10061	2	5031	2.760273E+05	68	4059	1.23930	0.296043
Uracil	1164	2	582	1.164013E+04	68	171	3.40069	0.039118
Glycerol	43114	2	21557	2.566513E+06	68	37743	0.57115	0.567563
Benzoate	312	2	156	2.421352E+03	68	36	4.37445	0.016325
Ornithine	299110	2	149555	1.247760E+07	68	183494	0.81504	0.446899
Choline	466	2	233	2.404914E+04	68	354	0.65873	0.520778
GABA	8604986	2	4302493	3.615796E+07	68	531735	8.09143	0.000704
Cytosine	20	2	10	1.065213E+03	68	16	0.63992	0.530480
CLA	1	2	0	2.548133E+00	68	0	12.96349	0.000017
caproleic	0	2	0	6.232829E-01	68	0	2.22775	0.115579
butyric	3	2	1	7.855513E+01	68	1	1.19826	0.308008
LN	1	2	0	3.823920E+00	68	0	6.45332	0.002715
LO	0	2	0	1.170105E+01	68	0	1.22987	0.298749
OL	15	2	8	5.307377E+02	68	8	0.96520	0.386063
SAT	31	2	15	6.192266E+02	68	9	1.69422	0.191406
Sterols	0	2	0	1.583282E-02	68	0	7.06198	0.001634
1,2-DG	6	2	3	2.605879E+01	68	0	7.79272	0.000897
TFAA	167200715	2	83600357	7.815880E+09	68	114939418	0.72734	0.486912
Propionate	0	2	0	2.839432E+01	68	0	0.18328	0.832941
Butyric	1	2	0	4.694759E+02	68	7	0.06932	0.933094
Galactose	6	2	3	8.734047E+02	68	13	0.24754	0.781422

Table S5. Cross validation ANOVA of the untargeted OPLS-DA model regarding cretan/non cretan origin depicted in Figure 6, right.

Cretan/Non cretan (OPLS-DA)	SS	DF	MS	F	p	SD
**Total corr.**	73	73	1			1
**Regression**	35.4142	10	3.54142	5.93599	2.91834e-006	1.88186
**Residual**	37.5858	63	0.596601			0.772399

Figure S1. Permutation diagram of the untargeted PLS-DA model regarding cretan/non cretan origin depicted in Fig. 6, right.

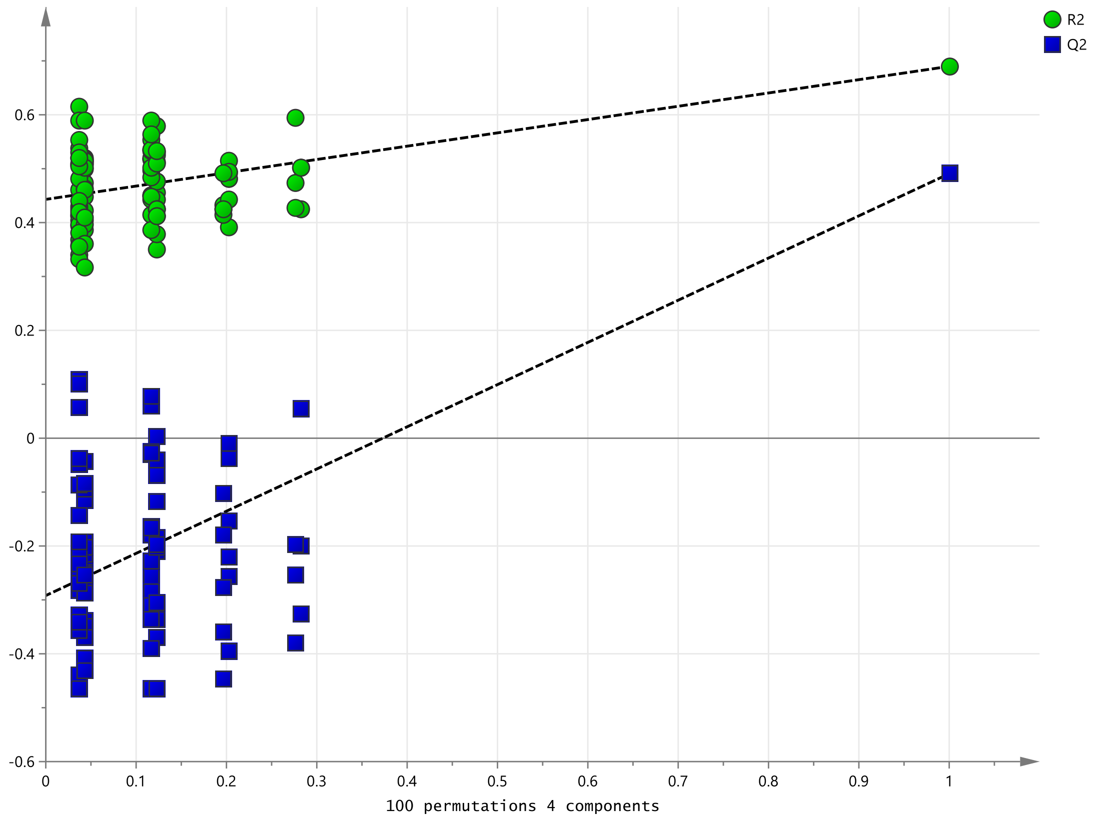

Figure S2. Aliphatic region of the ^{1}H-^{13}C multiplicity-edited gHSQC 2D NMR spectrum of the polar extracts of graviera cheese at a ^{1}H frequency of 500 MHz, blue contours represent CH/CH_3 protons, green contours represent CH_2 protons.

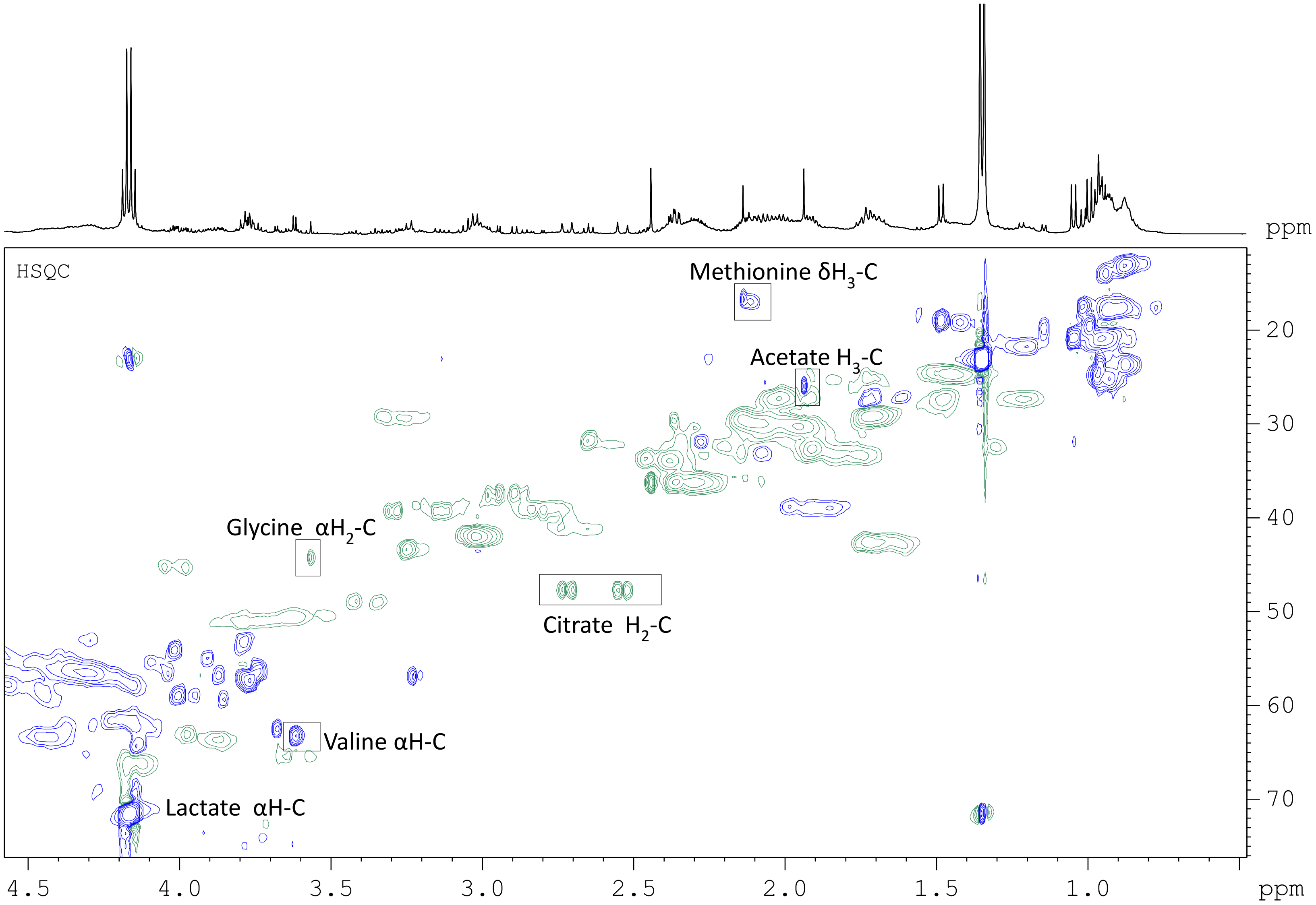

Figure S3. Aromatic region of the ^{1}H-^{13}C multiplicity-edited gHSQC 2D NMR spectrum of the polar extracts of graviera cheese at a ^{1}H frequency of 500 MHz, blue contours represent CH/CH_3 protons, green contours represent CH_2 protons.

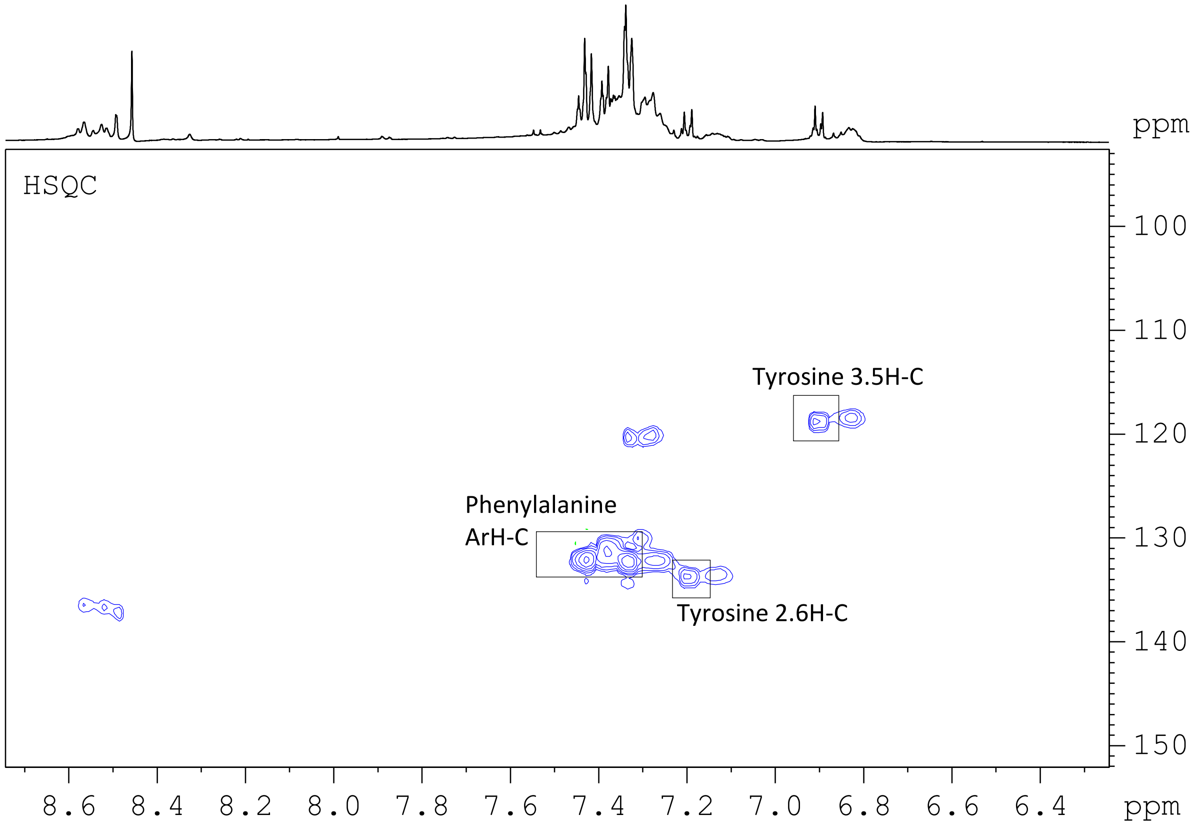

Figure S4. Aliphatic region of the long range ^{1}H-^{13}C gHMBC 2D NMR spectrum of the polar extracts of graviera cheese at a ^{1}H frequency of 500 MHz.

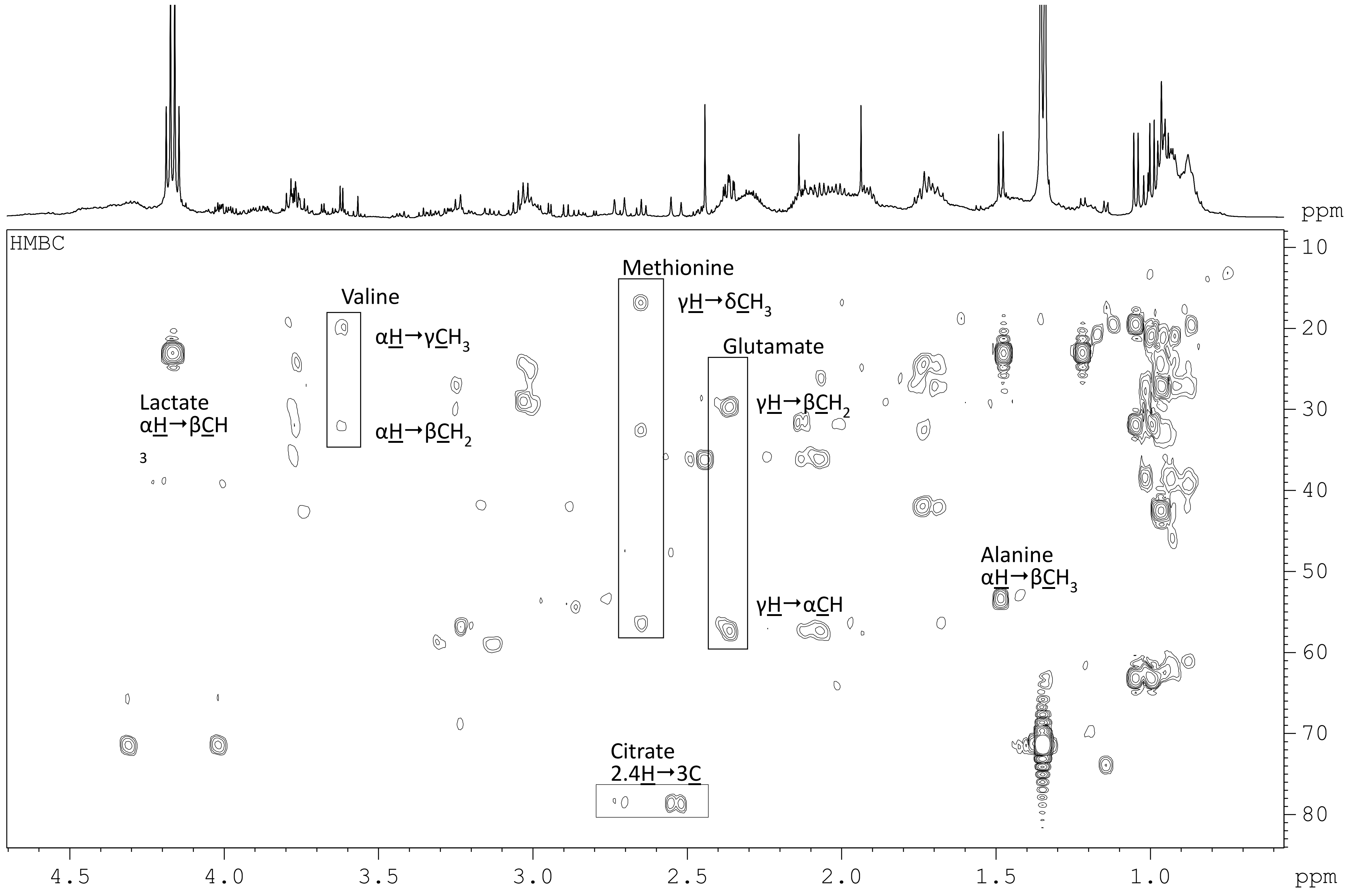

Figure S5. Aliphatic region of the ^{1}H-^{1}H gTOCSY 2D NMR spectrum of the polar extracts of graviera cheese at a ^{1}H frequency of 500 MHz.

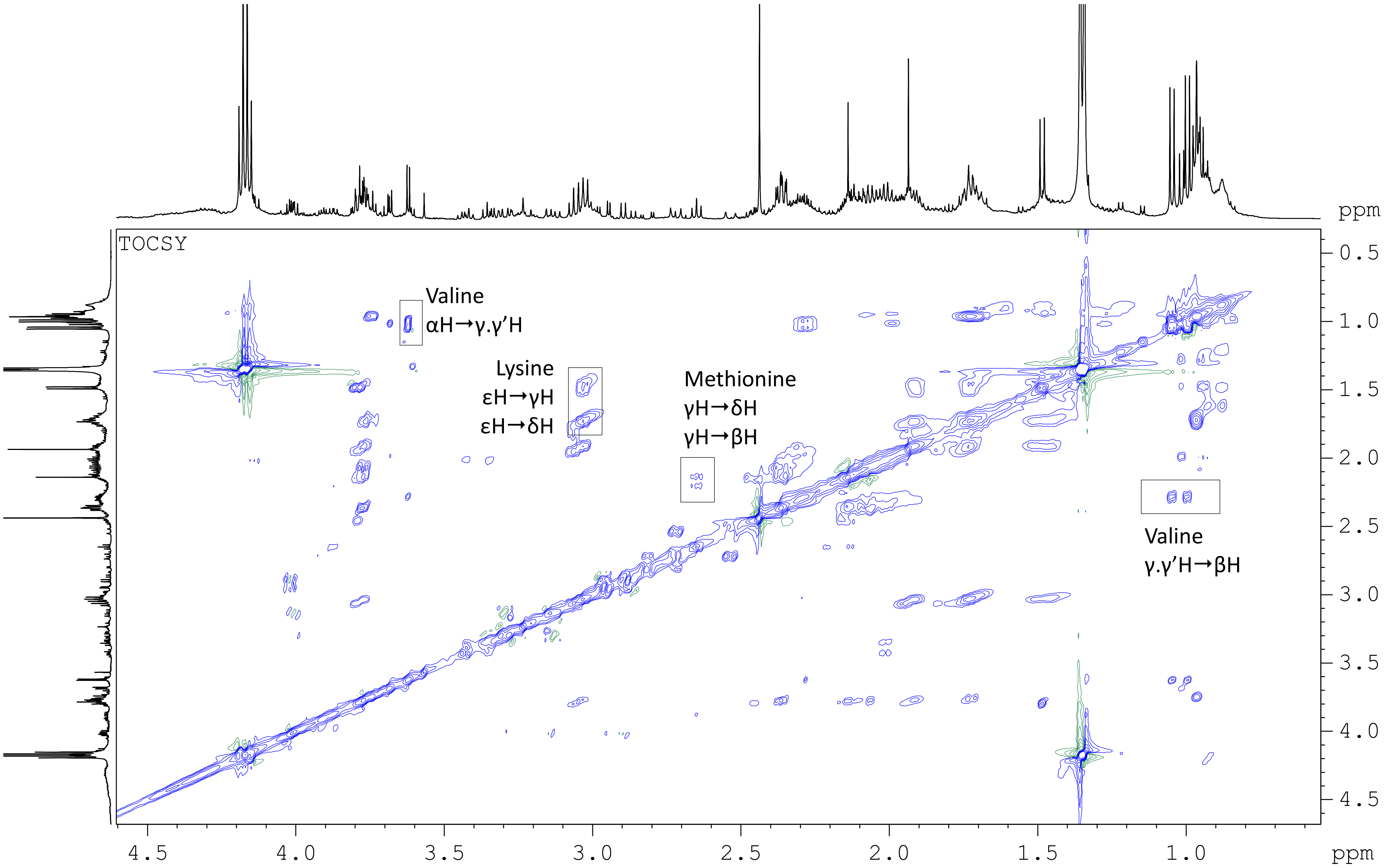

Supplement: Supplementary file 1 [file molecules-28-05488-s001.zip › molecules-2446508-supplementary.pdf]
